# Menstrual Cycle and Athletic Status Interact to Influence Symptoms, Mood, and Cognition in Females

**DOI:** 10.1186/s40798-025-00924-8

**Published:** 2025-10-10

**Authors:** Flaminia Ronca, Evelyn Watson, Isabel Metcalf, Benjamin Tari

**Affiliations:** 1 Institute of Sport, Exercise & Health, Division of Surgery and Interventional Sciences, University College London, London, UK; 2 College of Life and Environmental Sciences, University of Exeter, Exeter, UK

**Keywords:** Female, Exercise, Executive function, Sport, Ovulation, Activity

## Abstract

**Background:**

There is growing interest in understanding if, and how, the menstrual cycle may affect physical and cognitive performance, particularly in the context of sport and physical activity. While hormonal fluctuations are often assumed to impact cognition, especially during menstruation, empirical evidence remains inconclusive. This study aimed to investigate whether cognitive performance, mood, and symptomology vary across menstrual cycle phases and whether these effects are influenced by athletic participation level.

**Results:**

Fifty-four females (18–40 years) categorised by athletic participation level (i.e., inactive, active, competing, elite) took part. At each key menstrual phase (menstruation / early follicular, late follicular, ovulation and mid-luteal), they completed a cognitive battery (attention, inhibition and spatial anticipation), and reported their mood and symptoms. Faster reaction times and fewer errors were committed during ovulation (*p* < .01), suggesting better overall performance. In contrast, reaction times were slower during the luteal phase (*p* < .01), but more errors were committed in the follicular phase (*p* = .01). Importantly, participants’ athletic level had a stronger effect on cognitive performance than phase, where inactive participants scored worse across tasks than their more active counterparts, and elite participants exhibited more significant fluctuations in cognition across phases. Mood and symptoms were worse during menstruation regardless of athletic level. However, while mood varied across phases it did not correlate with cognitive performance. Of note, participants perceived that their symptoms negatively impacted their cognitive performance during menstruation, but there was no evidence of any objective detriment to cognitive performance during this phase neither on reaction times nor errors on any task.

**Conclusion:**

These findings suggest the existence of mild cognitive fluctuations throughout the menstrual cycle, albeit with high individual variability, and which are incongruent with symptomology. Opposing results between perceived and measured performance challenge common assumptions about menstruation-related performance, and highlight the importance of addressing societal biases in female sport and health. The stronger effects of athletic engagement on cognitive performance, rather than phase, reinforce the cognitive benefits of an active lifestyle.

**Key Points:**

This study aimed to investigate cognitive fluctuations in eumenorrheic females recruited from a range of activity levels.

We demonstrate mild cognitive fluctuations throughout the menstrual cycle, which are incongruent with symptomology.

And reinforce the importance of physical activity for supporting cognitive performance.

**Supplementary Information:**

The online version contains supplementary material available at 10.1186/s40798-025-00924-8.

## Introduction

The recent growing interest in female health research has highlighted the importance of considering the potential interactions between the menstrual cycle and physical activity. Participation and investment into female sport have grown in recent years [1], but there is still a dearth of research focused on understanding female physiology in the context of sport and exercise. As of 2021/22, only 6% of sport science literature [2], and 0–8% of sport nutrition literature, depending on the supplement [3], investigated all-female participant samples; indeed, female-specific studies were published eight times less often than male-only studies [3]. It is likely not a coincidence that many exercise science studies have purposefully opted to focus on all-male samples to avoid the presumed confounding effects of hormonal fluctuations present in females [4, 5]. Despite this assumption, few authors have attempted to understand if such effects were present in their data; in Smith et al.’s sport nutrition review [3], only 14% of 1,826 papers that included either mixed samples, or all-female samples, attempted to categorise menstrual status, and of these, only three papers used best-practice methods to do so. Given the pervading belief that the phases of the menstrual cycle might relate to physiological, physical, and cognitive processes in sport [6–8], the field requires a better understanding of whether and how the phases of the menstrual cycle might impact participant outcomes.

In naturally menstruating females (i.e., not using hormonal contraception), the eumenorrheic menstrual cycle consists of a fluctuation of hormones over a 28-day cycle (range 21–35 days). Key hormones which fluctuate during this period include oestrogen, progesterone, luteinising hormone (LH), and follicle stimulating hormone (FSH). The former two hormones drive the main phases of the menstrual cycle which include an early follicular phase (i.e., days of menstruation, with low levels of oestrogen and progesterone) followed by the late follicular phase (i.e., oestrogen rises and peaks prior to ovulation), ovulation (i.e., the release of an egg from an ovary), and the luteal phase (i.e., a second, smaller rise in oestrogen and peak progesterone) [9].

During the menstrual cycle, females have tended to self-report worse perceived physical and cognitive performance in relation to specific phases, particularly during menstruation [6–8, 10, 11]. Such perceptions have been surveyed in team and individual sports, where athletes self-report diverse patterns in symptom and performance changes, further highlighting the strong individuality of how females experience their own cycles [6–8, 10, 11]. Injury risk has been suggested to also be affected [12], with a higher prevalence of musculoskeletal injuries and concussions being reported during the luteal phase, particularly in team sports [13, 14]. Finally, different concentrations of ovarian hormones across the phases of the menstrual cycle have been associated with changes to several cognitive domains, including those specifically relevant to sporting performance and injury prevention [15]. Although findings are still inconclusive [16–18], the papers that do report an effect have suggested worse cognitive performance during latter phases of the cycle [16–18]. Theoretical explanations of such outcomes include oestrogen’s excitatory effects on the cerebral cortex [19, 20], particularly across key executive-function-related regions within the prefrontal cortex [10, 21], and progesterone’s contrasting inhibitory effects [20, 22]. Additional complex interactions between functional connectivity and regional receptor availability in the hippocampus, amygdala, and prefrontal cortex [23–25] have also been posited. Results of a recently published proof-of-principle paper reported slower reaction times in sport-related cognitive processes during latter phases of the cycle and better overall performance during menstruation [15]. These results are especially interesting given participants perceived worse performance during this phase. While these initial exploratory findings suggest a potential effect of the menstrual cycle on attention, inhibition, and spatial cognition, the high variability in results reported in the proof-of-principle paper [15] and in the literature [16, 18] necessitate further consideration with the implementation of more robust cycle-tracking methods [16]. Accordingly, more work is needed to better understand if, and how, particular phases of the menstrual cycle might influence sport-related elements of cognitive performance, and whether these are congruent with females’ self-reported perceptions of inconsistent performance [6–8].

Furthermore, in the context of female sport, participation in physical exercise at varying athletic levels is known to impact the degree of hormonal fluctuations across the cycle [26]. Given that physical fitness and sport participation have been consistently demonstrated to support executive functioning [27–30], exercise participation could further influence variability in cognitive performance across phases. This study therefore aimed to further investigate the putative cognitive fluctuations in regularly and naturally menstruating females, and to explore potential interactions between cognitive function and athletic participation level. We hypothesised that phases in which oestrogen levels are higher (i.e., ovulation) would exhibit faster reaction times but with more errors, whereas phases in which progesterone is the predominant hormone (i.e., luteal phase) would exhibit slower reaction times. Moreover, given the established beneficial effect of sport participation on cognitive function, we anticipate a lower variability across phases in active groups.

## Methods

### Participant information

Fifty-four naturally menstruating females (i.e., not using hormonal contraceptives) were recruited to take part in this study via word of mouth and social media. The sample size employed here was deemed suitable to conduct a repeated measures ANOVA to derive between- and within-subject effects and interactions for four groups (power = 0.95, effect size = 0.25). All participants were informed of the study’s purpose to “explore whether cognitive processes change across the menstrual cycle” when recruited, and (1) were aged between 18 and 40 years; (2) self-reported a regular menstrual cycle lasting between 21 and 35 days; (3) self-reported that they did not experience regular spotting outside of menstruation; (4) had tracked their period for at least three months prior to beginning the study; (5) were not taking any hormonal contraceptives or other form of hormonal medication in the previous three months; and (6) had not been pregnant or breastfeeding in the previous six months. Participants self-reported their participation in regular or competitive sport Individuals were categorised as “inactive” if they reported not taking part in any form of regular physical activity. Those who participated in physical activity at least twice a week or were competing below club level were categorised as “active”. Participants were categorised as “competing” if they were involved in club level sporting competition (of any sport), and participants who competed at national or international levels were categorised as “elite”. According to the framework described by McKay and colleagues, ‘inactive’ would correspond to ‘sedentary’, ‘active’ to ‘recreationally active’ and ‘competing’ to ‘highly trained’, where objective and more detailed information would be required to confirm such classifications [31]. Participants provided informed written consent of a protocol approved by the University College London Research Ethics Committee (13985/007). All study activities were undertaken in accordance with the most recent iteration of the Declaration of Helsinki.

### Study design and cycle phase categorisation

**Fig. 1:**
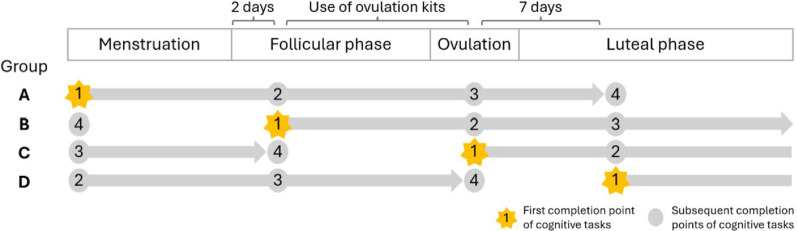
Graphical representation of study timelines indicating the start- and endpoints for each study group

Participants first attended the laboratory to collect urinary kits, and to run through a practice trial of the cognitive test battery, and were provided a link to the online cognitive battery hosted on the Gorilla Experiment Builder [32] for home use. The battery took 10–15 min to complete and included a simple reaction time task, a sustained attention task (i.e., No-Go/Go), an inhibition task (i.e., Go/No-Go), and a spatial timing anticipation task. Participants were instructed to complete this assessment battery four times over the course of one cycle in a counterbalanced randomised order: (1) the first day of bleed (i.e., menstruation/early follicular phase); (2) two days after bleeding had ceased (i.e., late follicular phase); (3) on the day when ovulation was detected (i.e., ovulation); and (4) seven days following ovulation (i.e., mid-luteal phase). In the interest of consistency, we defer to the classification terminology referenced in Elliot-Sale et al.’s [33] opinion article when making these classifications. Participants were randomly allocated into one of four study groups wherein one quarter of the study sample began cognitive testing at stages 1, 2, 3, or 4, respectively, followed by testing at the subsequent three timepoints (i.e., Group A: 1–2 – 3–4; Group B: 2–3 – 4–1; etc.) (see Fig. 1 for study timelines). They were instructed to complete the test battery on their own computers at home, in a quiet environment void of distraction, in the afternoon, at the same time each week, and not immediately after exercise. They were asked to not modify their daily routines in any way over the course of this study.

Participants were provided at-home urinary ovulation kits (Clearblue Advanced Digital Ovulation Test, Swiss Precision Diagnostics GmbH (SPD, Petit-Lancy, Switzerland) to approximate their phases over the course of the study. Participants were instructed to use the ovulation test kits every morning, starting two days after bleed cessation (timepoint 2 above), until ovulation was detected. The ovulation day cognitive test was then conducted that same afternoon.

### Cognitive battery, mood and symptom questionnaires

The battery of cognitive tests implemented in this study has been previously validated and used in cohorts of varying ages. It has also been demonstrated to be sensitive to fluctuations in the menstrual cycle [15], and related to physical activity levels [34]. Cognitive tests were selected based on their relevance to sporting processes, as well as their potential sensitivity to changes in the menstrual cycle. A more complete rationale of why these specific tests were chosen has already been published [15]. Key components of each task are briefly outlined below, and extensive details on the selection, development, validation and processing of the tasks can be found in Ronca et al. [15].

The simple reaction time task required participants to press the spacebar as quickly as possible when an illustration of a smiling or winking face appeared on the screen (40 trials). The sustained attention task required participants to press the spacebar when presented with a winking face (10 trials) but to withhold pressing the spacebar when presented with a smiling face (20 trials) (i.e., 30 trials total). In contrast, the inhibition task was used as an assessment of inhibitory control and required participants to press the spacebar when they were presented with a smiling (62 trials), but not a winking face (12 trials) (i.e., 74 trials total). Reaction times for each task were calculated as the mean reaction time of all correct responses slower than 125 ms as per standard practice [35]; where any presses that occurred before 125 ms were counted as commission errors. Commission errors were additionally calculated as the total sum of presses during inter-trial intervals for the simple reaction time task, and during No-Go stimuli for the sustained attention and inhibition tasks. Reaction times and commission errors for each of these three tasks were factor analysed using varimax rotation to derive a single Composite Reaction Time score and a Composite Commission Error score.

Finally, the timing anticipation task involved reacting to two footballs moving toward each other across the computer screen. Participants were required to press the spacebar when the balls were expected to contact each other. For one half of trials, the balls were hidden from view after movement began and were invisible for the remainder of the trial. Fourteen visible trials and 15 hidden trials were completed (i.e., 29 total trials). Timing error on this task was calculated as the absolute mean difference (ms) between the moment the spacebar was pressed, and the actual moment the two balls made contact on the screen. Timing error variability for this task was calculated as the standard deviation of all timing errors.

To quantify mood, the Burgess Brief Mood Questionnaire [15] was used to monitor participants’ ratings of 10 mood statements on a Likert scale of 1 to 7 (see Supplementary material). This scale was selected as it has been previously shown to be sensitive to both changes in mood state following different forms and intensities of physical activity [Jackson et al. under review], and to different phases of the menstrual cycle [15]. Scale items included “I feel alert” and “I feel calm”. Factor analysis with polychoric correlations derived two measures: “Drive” (i.e., feeling energetic and focused) and “Serenity” (i.e., feeling calm and content) [15].

Finally, participants were provided with lists of cognitive symptoms (e.g., headaches, brain fog), physical symptoms (e.g., back pain) commonly experienced throughout the menstrual cycle, in line with previously validated methods [36, 37] (see Supplementary material). Participants were asked to select all symptoms they were currently experiencing. These were summed for analysis.

### Dependent variables and statistical analysis

Mood dependent variables included factor scores of derived Drive and Serenity, whereas cognitive and physical symptom severity variables included the total count of symptoms reported by participants. Cognitive dependent variables for the simple reaction time task, inhibition task, and sustained attention task included mean reaction time (i.e., time taken in ms to respond to a stimulus after presentation) and Composite Commission Errors (i.e., the number of incorrect responses in relation to the presented stimuli). Dependent variables for the timing anticipation task included mean timing error (i.e., time in ms by which participants had mis-judged the collision of the football icons) and mean timing error variability (i.e., the standard deviation of timing errors).

All analyses were conducted in R Studio [38]. All variables were tested for normality through visual inspection and a Shapiro-Wilk test. Where variables were not normally distributed, they were either BoxCox transformed or assessed via non-parametric tests where appropriate. All dependant cognitive and mood variables were assessed via 4 (group: inactive, active, competing, elite) by 4 (phase: menstruation/early follicular phase, late follicular phase, ovulation, mid-luteal phase) repeated measures ANOVA (α = 0.05). Significant interactions were decomposed via simple-effects (i.e., Bonferroni corrected t-test). A chi-square test was conducted on the total number of symptoms reported to test for phase effects. ANCOVAs controlling for Drive and Serenity, and exploratory correlations by phase, were conducted to test for effects of mood on cognitive performance by cycle phase. Alpha levels were set to *p* < .05 for statistical significance. Effect sizes were interpreted as small (η_p_² = 0.01 − 0.05), moderate (0.06 − 0.13) or large (>0.14).

## Results

### Participants

A total of 165 participants volunteered to take part in the study. Of these, 60 took part and 54 participants (Age = 28 ± 6 years, BMI = 23.6 ± 4.4, average cycle length = 29 ± 2 days) provided a full set of results. Reasons for dropping out included loss of contact with the study research team (*n* = 3), forgetting to complete the study assessment battery at the specified time (*n* = 2), and failure to identify day of ovulation using the urinary kits by the participant (*n* = 1). There were no significant differences in age, BMI or cycle length between groups (Table 1). Participants were divided into four athletic participation groups according to self-reported levels. For those who participated in sports, 20 active participants recreationally took part in: football = 4, cycling = 3, swimming = 3, tennis = 3, rowing = 2, weightlifting = 2, rugby = 1, athletics = 1, martial arts = 1; 10 competing participants took part in: football = 5, rowing = 3, basketball = 1, swimming = 1; and 12 elite participants were part of a same elite football team.

**Table 1:** Demographic information of study participants following categorisation by athletic level

	Inactive (*n* = 12)	Active (*n* = 20)	Competing (*n* = 10)	Elite (*n* = 12)
Age	28 ± 7	29 ± 6	30 ± 5	24 ± 5
Height (cm)	166 ± 4	168 ± 8	171 ± 6	168 ± 8
Weight (kg)	63 ± 14	60 ± 8	79 ± 20	67 ± 14
BMI	22.9 ± 4.7	21.7 ± 2.4	26.7 ± 5.9	23.6 ± 3.6
Cycle length (days)	29 ± 3	29 ± 2	28 ± 1	30 ± 2

### Cognitive function

**Fig. 2:**
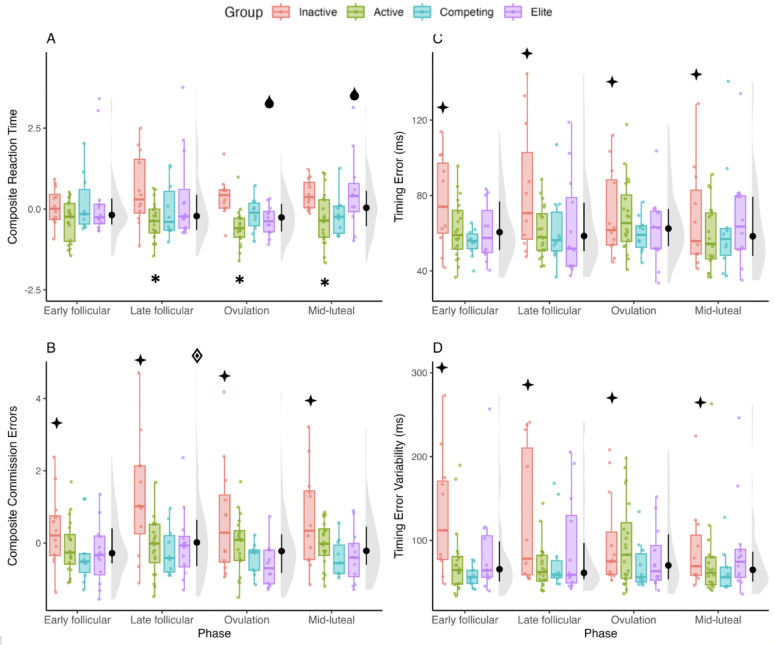
Group median ± IQR for Composite Reaction Time (**A**), Composite Commission Error (**B**), Timing Error (**C**), and Timing Error Variability (**D**). Coloured box plots indicate values divided by athletic group; grey density plots to the right of the box plots indicate overall group distribution per phase, with median and IQR in black. Significant interaction effects for phase, within the elite group (*p* < 0.05); Significant main effect of phase (*p* < 0.05); Significant main effect of group (*p* < 0.05); * Significant interaction effects for group, within phases (*p* < .05)

On the attention and inhibition battery, participants exhibited faster reaction times and committed fewer errors during ovulation, whereas reaction times were slower during the mid-luteal phase, but more errors were committed during the late follicular phase (Fig. 2). As for athletic level, participants who were categorised as “inactive” exhibited slower reaction times and committed more errors than the other groups.

Specifically, Composite Reaction Time (Fig. 2A) exhibited a main effect of phase, *F*(3,150) = 4.53, *p* = .005, η_p_^2^ = 0.08, where scores were faster during ovulation compared to the mid-luteal (*p* = 0.003) and late follicular (*p* = .04) phases; and a main effect of group, *F*(3,50) = 3.80, *p* = .02, η_p_^2^ = 0.18, where active participants had faster reaction times than the inactive group (*p* =0.02). There was also a group by phase interaction, *F*(9,150) = 2.95, *p*= 0.003, η_p_^2^ = 0.16, which revealed that only elite participants had faster reaction times during ovulation compared to the mid-luteal phase (*p* < .0.001), the late follicular phase (*p* = .0.01) and early follicular phase (*p* =0.03), and slower reaction times during the mid-luteal phase compared to the early follicular phase (*p* < .0.03). During the mid-luteal phase, elite participants also exhibited slower reaction times than the competing (*p* = 0.001) and active (*p* = .0.02) groups. In addition, active participants exhibited faster reaction times than inactive ones during the late follicular (*p* = .0.02) and ovulation (*p* = 0.02) phases.

For context and interpretability, specific reaction times during the sustained attention task and inhibition task averaged 482 ± 81 ms and 390 ± 69 ms, respectively, during ovulation. This is compared to 531 ± 138 ms and 430 ± 127 ms during the mid-luteal phase across the study sample indicating an overall group-level difference of 50 ms and 40 ms, respectively, between these two phases. In contrast, average reaction time during the sustained attention task and inhibition task totalled 581 ± 123 ms and 426 ± 69 ms for the inactive group across phases, compared to 475 ± 95 ms and 375 ± 87 ms for the active group. Thus, results revealed a difference by athletic level of 105 ms and 50 ms for the two tasks, respectively. For a full table of individual task and group reaction times, and related statistical analyses, please see Supplementary material.

For Composite Commission Errors (Fig. 2B), there was a main effect for phase, *F*(3,150) = 3.85, *p* = .0.01, η_p_^2^ = 0.07, where fewer errors were committed during ovulation compared to the late follicular phase (*p* = .0.01). There was also a main effect for group, *F*(3,50) = 3.48, *p* = .02, η_p_^2^ = 0.17, where inactive participants committed more errors than the elite (*p* = 0.03) and the competing (*p* = .0.05) groups. No phase by group interactions were found.

Results of the spatial anticipation task produced no main effect of phase, *F*(3,50) = 0.68. *p* = .0.06, η_p_^2^ = 0.01, but did produce a main effect for group for both timing error, *F*(3,50) = 3.46, *p* = .0.02, η_p_^2^ = 0.18, and for timing error variability, *F*(3,50) = 4.42, *p* = .0.008, η_p_^2^ = 0.21, respectively (Fig. 2C and D). Participants categorised as inactive had larger timing error (87 ± 60 ms) than their active (64 ± 20 ms) (*p* = .0.03) and (60 ± 19 ms) (*p* = .0.04) counterparts; and more variable (138 ± 130 ms) than the active (78 ± 44 ms) (*p* = .0.007), competing (80 ± 79 ms) (*p* = .0.03) and elite (87 ± 53 ms) (*p* =  0.05) groups.

### Mood and symptoms

**Fig. 3:**
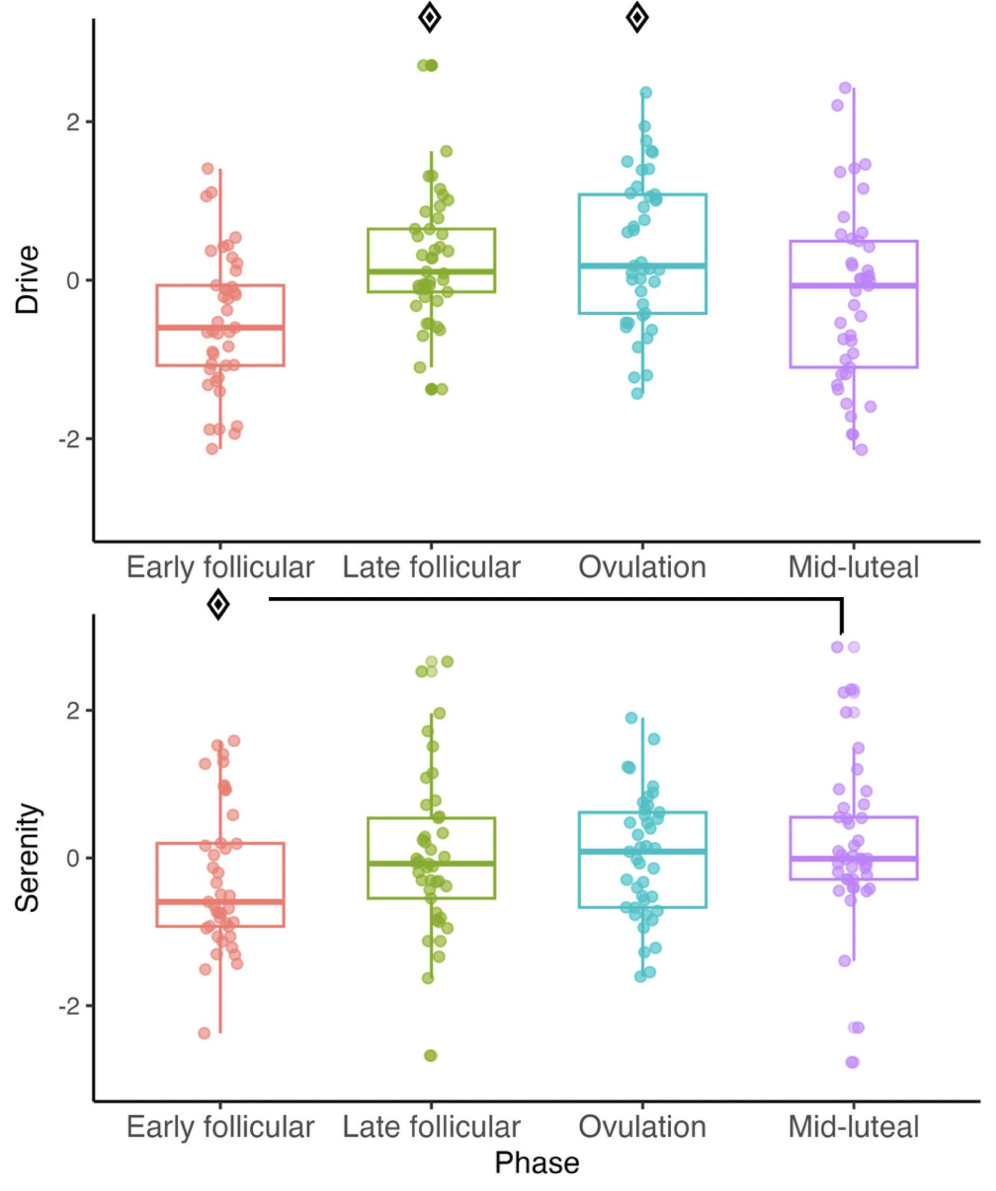
Participant-specific and group mean data for mood constructs of Drive (Top) and Serenity (Bottom) by cycle phase across all athletic groups. Significant phase effect (*p* < .05)

Drive yielded a main effect of phase, *F*(3,112) = 8.30, *p* < .0.001, η_p_^2^ = 0.18, but not group, *F*s(3,37) = 0.37, *p* > .0.06, η_p_^2^ = 0.03, where participants reported higher Drive during both ovulation and the late follicular phase compared to the early follicular phase (*p* < .001) and the mid-luteal phase (*p* < .05). Serenity also demonstrated a main effect of phase, *F*(3,112) = 3.07, *p* = .0.03, η_p_^2^ = 0.13, but not group, *F*(3,37) = 0.55, *p* > 0.6, η_p_^2^ = 0.03, with higher Serenity during the mid-luteal phase compared to the early follicular phase (*p* = .0.02) (Fig. 3).

**Fig. 4:**
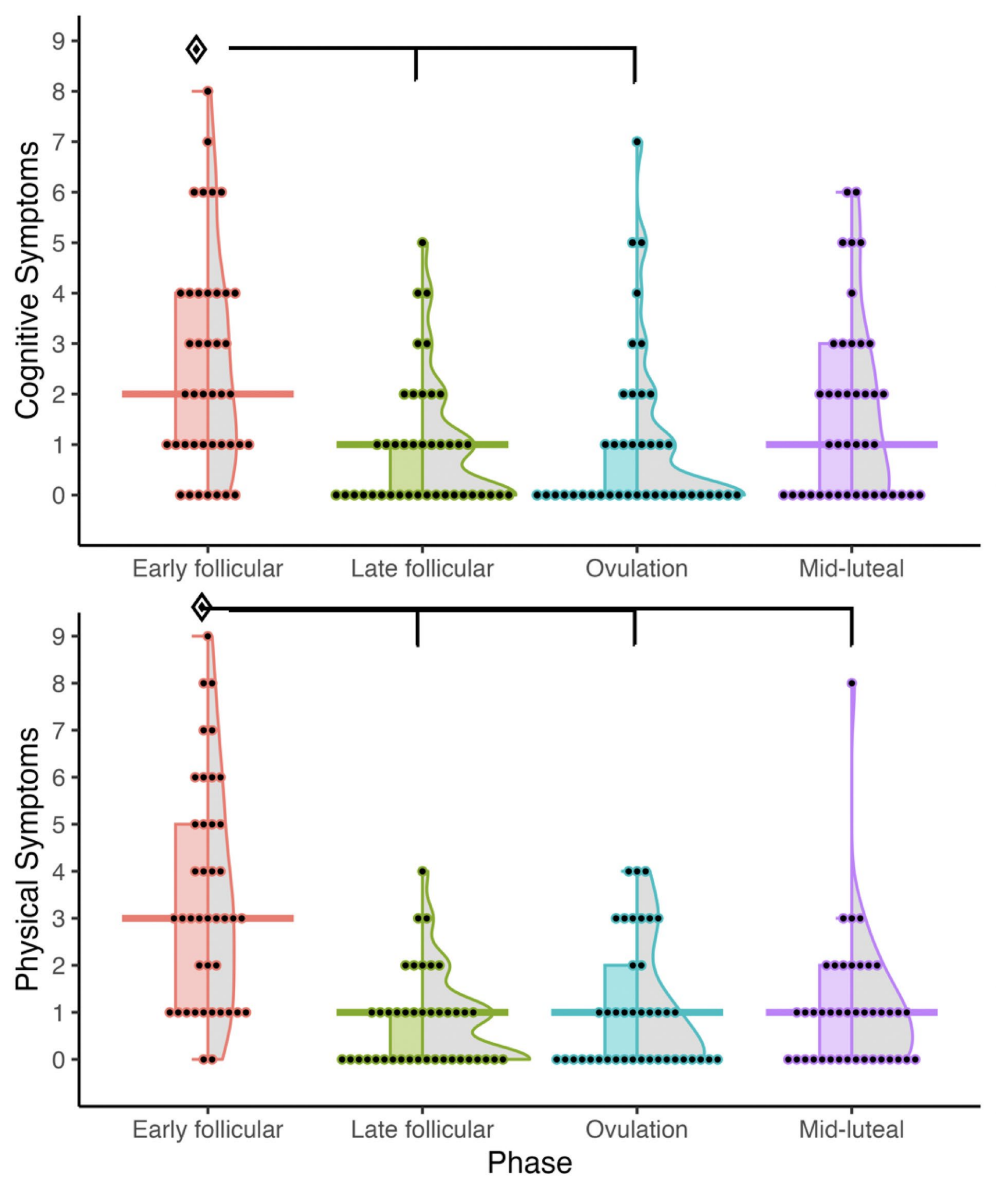
Total (n) cognitive and physical symptoms experienced by participants according to cycle phase; mean is denoted by a solid horizontal line. Significant phase effect (*p* < 0.001)

For self reported symptoms, cognitive symptoms demonstrated a main effect of phase, *F*(3,111) = 10.36, *p* < .0.001, η_p_^2^ = 0.21, but not for group, *F*(3,37) = 0.85, *p* > 0.4, η_p_^2^ = 0.06. A higher total number of cognitive symptoms were reported during the early follicular phase (3 ± 2) compared to all other phases (ps < 0.001). Similarly, there was a main effect of phase for physical symptoms, *F*(3,111) = 35.32, *p* < .0.001, η_p_^2^ = 0.44, but no effect for group, *F*(3,37) = 1.13, *p* > 0.4, η_p_^2^ = 0.08, where a higher number of physical symptoms were reported during the early follicular phase (3 ± 2) compared to the late follicular phase and ovulation (ps < 0.001) (Fig. 4). Full detail on the number of participants who reported experiencing each specific symptom is included in the supplementary material.

### Perceived performance

Participant’s perception of cognitive performance was reliably related to cycle phase (X^2^(6) = 21.24, *p* < .002), where 55% of participants (*p* = .03) believed that their symptoms were negatively impacting their cognitive performance during the early follicular phase. This effect did not differ by group and there was no effect of perceived impact on any repeated measures ANCOVAs conducted on Composite Reaction Times, Composite Commission Errors, timing error nor timing error variability. Exploratory analyses also yielded no significant correlations between mood and cognitive performance in any phase. Together, these results suggest that the effect of phase on cognitive performance was independent of changes in mood, and symptoms experienced.

## Discussion

This study sought to examine whether performance on sport-related cognitive domains varied according to menstrual cycle phase, and whether these effects differed by athletic participation level. Overall, attention and inhibition task performance were best during ovulation, particularly in elite athletes. Inactive participants generally scored poorer than other groups regardless of phase, producing slower reaction times, as well as more commission errors and greater spatial timing anticipation errors. Finally, participants reported worse mood, and cognitive and physical symptoms during menstruation despite cognitive performance not being worse at this time. The results highlighted that menstrual cycle phases were differentially associated with changes in cognition and mood, that these changes did not align with participant’s perceptions, and that participant activity level interacted with cognitive performance but not mood.

### Cognitive performance and menstrual phases

Composite scores of the attention and inhibition battery indicated faster reaction times and fewer errors on the day of ovulation compared to other phases. In contrast, Composite Reaction Times were slower during the mid-luteal phase compared to both ovulation and the early follicular phase, but Composite Commission Errors were higher during the late follicular phase. The slower reaction times observed during the mid-luteal phase are in line with those described in the prior proof-of-principle paper by Ronca and colleagues [15] and, while the added insight into improved performance during ovulation provides a more comprehensive picture of such effects. Furthermore, there was no evidence to any detriment to cognition during the early follicular phase.

The better overall performance during ovulation (i.e., faster reaction times and fewer commission errors) could be explained by the effects of fluctuating oestrogen and progesterone levels on cortical activity over the course of the menstrual cycle. For example, increased oestrogen concentrations (either via oestrogen replacement therapies or during the menstrual cycle) have been shown to increase neural activity in the prefrontal cortex [39–41], as well as alter neurotransmitter levels and cortical morphology [42]. Oestrogen, which typically peaks before, ovulation is also purported to benefit executive control, as previous work has demonstrated excitatory effects of oestrogen on cortical regions which support cognition [19, 20]. Specifically, Smith et al. [22] demonstrated excitatory effects of oestradiol during ovulation, which may benefit some cognitive processes and subjective state of alertness as demonstrated here. Work investigating the effects of oestrogen on the dorsolateral prefrontal cortex (DLPFC) (i.e., involved in higher-order cognitive control; [21]) have demonstrated similar positive results [43]. These results intimate that oestrogen concentration could be a key factor when considering the performance of higher-order cognitive functions. We note that mood regulation has also been linked to areas of the prefrontal cortex [44] whose rate of activity fluctuates according to hormone concentrations. In fact, the higher Drive (i.e., feeling of alertness and energy) observed here during the late follicular phase and ovulation are in line with previous research [12, 15] and might be attributed to the increase in oestrogen that occurs mid-cycle [9] and the concomitant increase in cortical activity [42].

In direct contrast, the slower reaction times observed during the mid-luteal phase could be related to the increase in progesterone that characterises it. Progesterone concentrations are low during ovulation but increase during the luteal phase, where its interaction with GABA_a_ receptor complexes [20] is thought to reduce cortical activity in regions responsible for cognitive control. For example, increased progesterone has been found to inhibit cortical excitability [20, 22] in regions responsible for anxiety (i.e., amygdala), memory, and executive function (e.g., hippocampus, fusiform gyrus, and prefrontal cortex) [45]. It is important to note that, in the current study, the mid-luteal phase exhibited slower reaction times but not more errors. In fact, despite progesterone’s dampening effects on cortical activity [45], its concentration has also been shown to support sustained attention in young females in the luteal phase [46], suggesting that slower processing speeds do not necessarily impact accuracy.

Finally, the divergence between slower reaction times in the mid-luteal phase, but more commission errors during the late follicular phase, point to a complex interaction between key hormones and brain function. One other study [47] has previously reported less efficient inhibitory control during a stop-signal test in the follicular phase compared to the menstrual (i.e. early follicular) and luteal phases, where the results correlated with oestradiol, but not progesterone, levels. It is possible that the interacting fluctuations of multiple hormones (oestrogen, LH, FSH) that occur concomitantly in the late follicular phase might possibly explain poorer inhibition during this period of adjustment, as observed by Colzato and colleagues [47]. However, we emphasise that, while urinary kits were used to identify ovulation in this study, hormonal levels were not tested, leaving much to speculation. Accordingly, although it is likely that hormone concentrations underpin cognitive fluctuations across the menstrual cycle, additional neurobiological interactions (e.g., with LH and FSH fluctuations) [48, 49] should also be considered in future works.

### The relationship between cognition and menstrual phase is driven by athletic level

When results were examined according to athletic level, inactive participants were found to generate slower reaction times, more commission errors and a larger timing anticipation error than their more active counterparts. This effect was consistent across all phases of the menstrual cycle. The fact that inactive participants performed in such a way is perhaps not surprising given the extensive literature which indicates that increased physical activity supports cognitive function (e.g., inhibition, impulsivity, executive function, processing speed, attention, memory) [50], with further evidence that this effect is also specific within a female-only sample [51]. Importantly, the difference in reaction time between inactive and active participants was greater than that observed between different phases of the menstrual cycle both in magnitude and in effect size. For example, inactive participants were 72, 94, and 45 ms slower than all other groups on the simple reaction time (η^2^_p_ = 0.16), sustained attention (η^2^_p_ = 0.21) and inhibition tasks (η^2^_p_ = 0.14), and committed 3.2 and 2.8 times more impulsive errors in the sustained attention (η^2^_p_ = 0.17) and inhibition tasks (η^2^_p_ = 0.18) during the same phase, all with large effect sizes (η^2^_p_ >0.14). In contrast, phase differences reached 12, 50, and 41 ms between ovulation and the mid-luteal phase for the three tasks respectively, with moderate to small effect sizes (η^2^_p_ = 0.02, 0.08, 0.08) (See supplementary material for detail). This highlights the significant role of lifestyle factors in supporting cognition, above intrinsic biological fluctuations.

Additional findings which warrant further attention, however, are the apparent U-shaped relationship between athletic level and reaction time demonstrated during the mid-luteal phase. That is, participants who were inactive or elite athletes demonstrated slower reaction times during the mid-luteal phase than those who were competing or active, and elite athletes demonstrated the greatest fluctuation in cognitive function between phases. The stronger phase effects in the elite group could be potentially related to a difference in hormonal profiles that have been reported in athletes [52]. In part, this could relate to greater hormonal responses that occur in response to training [26], but also to the broader picture of the female athlete triad and relative energy deficiency [53, 54], as menstrual cycle disruptions are known to occur in elite athletes of varied sports [55, 56], sometimes resulting in functional hypothalamic amenorrhoea (i.e., the cessation of normal menstrual cycles) in more extreme cases [53]. Importantly, such effects have been shown to influence cognitive performance in athletes experiencing amenorrhea [57]. It might be that a potential milder exercise-related dysregulation in the elite group could be responsible for the cognitive outcomes observed here. Although females recruited in this study self-reported no cycle irregularities, hormone concentrations were not quantified, thus the broader metabolic impact of elite training on cognitive outcomes should be considered with caution and investigated further.

### An incongruent relationship between mood, perceived performance, and cognitive function

Mood and symptomology were related to menstrual phase wherein participants reported lower Drive and Serenity and experienced more cognitive and physical symptoms during the early follicular phase. Of note, 55% of participants perceived that these symptoms were negatively impacting their cognitive performance on their first day of bleed, but perceived and objective performance were unrelated. Participants’ cognitive performance was not negatively impacted during menstruation, and neither mood nor symptom prevalence were correlated with cognition at any cycle stage. This incongruence between perceived and actual cognitive performance is in line with findings demonstrated previously [15] and indicates a pervasive bias for females to associate cycle-related symptomology and mood with cognitive function. Indeed, athletes have often self-reported that menstruation negatively affects their performance [6, 10, 11]. For example, studies involving elite track and field and rugby athletes found 77% and 67%, respectively, believed their menstruation-related symptoms impaired their performance [58, 59]. We note, however, that Olympic-medal-winning performances have occurred across all phases of the menstrual cycle [60]. Moreover, subjective performance perception and objective performance do not align [8], and no differences have been reported in maximum oxygen consumption (V̇O_2max_) or peak treadmill velocity between phases [61]. Taken together, there appears to be an dissonance between actual and perceived cognitive and physical performance during menstruation that warrants further attention in practical settings, and could form the basis of supportive discussions in sports coaching (see also [6, 62, 63]).

### Study limitations

First, although hormonal tracking was conducted via at-home urinary testing, it should be noted that testing did not identify exact hormonal profiles across participants’ cycles. Furthermore, the data were based on a single menstrual cycle per participant (or two adjacent cycles), which restricted our ability to capture intra-individual variability. This is salient given that our results cannot be directly associated with individual profiles of oestrogen and progesterone fluctuations. Our results might also be attributable to additional hormonal fluctuations which cannot be described in the current body of work. Future work should seek to employ continuous hormonal testing across the cycle, aiming for a broad spectrum of assays.

Second, athletic participation level was self-reported, and the sample was not large enough to stratify by type of activity. Given the well-defined interaction between physical activity and cognition [50], future studies should consider obtaining objective measures of activity levels and physical fitness by modality (e.g., aerobic, strength, etc.) and skill domain (e.g., ball sports, endurance sports, artistic sports, etc.).

Third, although the start phase was randomised and counterbalanced across participants, we recognise that an order effect exists within the study design. It would have been logistically unfeasible to ensure counterbalancing across phases, given that these will always occur in sequence. This effect was mitigated by ensuring that an equal number of participants started the cognitive tests in each of the four selected timepoints.

Last, the online platform implemented for the cognitive testing supported inclusive participant recruitment which greatly facilitated study completion. However, it is important to consider that the cognitive battery presented here was self-administered and therefore the environment could not be controlled by the research team. Furthermore, the battery only tested a few key domains, making our results less generalisable across other cognitive functions. Additional higher-order and basic cognitive assessments should also be employed in future works to provide a more comprehensive picture of how hormonal fluctuations might relate to cognitive processes.

We also wish to highlight that our study sample did not include female participants who were actively using hormonal contraceptives as a comparator. Hormonal concentrations and their fluctuations are altered according to type of contraceptive used and this should be considered in future research.

### Significance of the results

The authors would like to emphasise three key points from these findings. First, although phase effects were present, these were small and subject to individual differences. Therefore, any clinical significance of these findings requires careful consideration and further investigation, and the causes for individual variability must be explored in future studies. Second, the effects of physical activity participation were much stronger than the effect of cycle phase. It is reassuring to see that the impact of exercise, which is more likely to be under one’s control, imparts a stronger effect than any putative effects of hormonal fluctuations. This highlights the significant positive role that lifestyle factors play in female cognition and should encourage a focus on engaging in physical activity. Third, the incongruence between perceived and actual performance during the early follicular phase provides further evidence against misconceptions about the effects of menstruation in female performance. We hope that these findings will help reshape cultural biases around the menstrual cycle and guide positive conversations between coaches and athletes.

### Conclusions

In summary, participants in this study demonstrated better cognitive performance on attention and inhibition tasks during ovulation, whereas slower reaction times and more errors were found during the luteal and follicular phases, respectively. Phase effects were stronger in elite athletes, while inactive participants performed consistently worse than active participants across phases. Importantly, statistical effects were greater by athletic participation level than by cycle phase. Finally, even though participants perceived that their symptoms negatively impacted their cognitive performance, there was no evidence of a detriment to cognition during menstruation compared to any other phase. Overall, these results provide evidence for cognitive fluctuations during the menstrual cycle, and an interacting effect of physical activity level which was stronger than cycle phase. Crucially, the changes in measured cognitive performance were incongruent with participants’ perceived performance. These results call for a reconsideration of societal biases on the presumed negative effects of menstruation on performance in females and highlight the importance of conducting a recreationally active lifestyle in supporting cognitive function. The high group and individual differences point to a possible heightened variability in those participating in elite-level sport which are worth investigating in more detail in order to better understand the effects of strenuous training on female health. Future research should consider investigating the mechanisms underlying these effects through accurate hormonal tracking and functional brain imaging and should investigate whether such effects are of any meaningful clinical significance.

## Supplementary Information

Below is the link to the electronic supplementary material.

Supplementary Material 1.

## Data Availability

The datasets used and/or analysed during the current study are available from the corresponding author on reasonable request.

## Abbreviations

LH: Luteinising hormone
FSH: Follicle stimulating hormone
DLPFC: Dorsolateral Prefrontal Cortex
