# Supplementary material for: Menstrual Cycle and Athletic Status Interact to Influence Symptoms, Mood, and Cognition in Females
Source: Sports Med Open. 2025 Oct 10;11:104. doi: 10.1186/s40798-025-00924-8 (PMC12511478; doi:10.1186/s40798-025-00924-8)
Supplement: Supplementary file 1 — Supplementary Material 1. [file 40798_2025_924_MOESM1_ESM.pdf]

Menstrual cycle and athletic status interact to influence symptoms, mood, and cognition in females

Flaminia Ronca[1], Evelyn Watson[1], Isabel Metcalf[2], Benjamin Tari[1]

[1]Institute of Sport, Exercise & Health, Division of Surgery and Interventional Sciences, University College London, London, United Kingdom

[2]College of Life and Environmental Sciences, University of Exeter, Exeter, United Kingdom

Burgess Mood Scale

Paul W. Burgess and Flaminia Ronca

Please tell us about your mood. How are you feeling right now?

1. Mentally, I feel energetic

Explanation of question: Someone who is "mentally energetic" is feeling ready for the rest of the day, and is generally feeling "up for it" and ready to go. Note that the question does not relate to how much energy physically you have, but refers instead to your *state of mind*.

1	2	3	4	5	6	7
Definitely not	Much less than usual	A little less than usual	About average	A bit more than usual	Definitely more than usual	A lot more than usual

2. I feel sad

Explanation of question: The question refers to how you feel *right now at this moment*.

1	2	3	4	5	6	7
Definitely not	Much less than usual	A little less than usual	About average	A bit more than usual	Definitely more than usual	A lot more than usual

3. I feel alert

Explanation of question: Right at this moment, do you feel like you are mentally sharp and fully aware of everything going on around you? Do you think you could react to things quickly?

1	2	3	4	5	6	7
Definitely not	Much less than usual	A little less than usual	About average	A bit more than usual	Definitely more than usual	A lot more than usual

4. I feel nervous

Explanation of question. Do you feel jittery at this moment? Signs of nervousness might include: raised heart rate, increased rate of breathing, a feeling of not wanting to sit still, sweating, and anticipation. (We don't mean nervous about how well you might perform your exercise, but nervousness more generally about life.)

1	2	3	4	5	6	7
Definitely not	Much less than usual	A little less than usual	About average	A bit more than usual	Definitely more than usual	A lot more than usual

5. I feel focussed

Explanation of question: Do you feel like you are clear about what you want to achieve right now and later on today? Do you feel "in the moment" and able to concentrate on what you are doing?

1	2	3	4	5	6	7
Definitely not	Much less than usual	A little less than usual	About average	A bit more than usual	Definitely more than usual	A lot more than usual

6. **I feel lethargic and lacking motivation**

Explanation of question: Do you feel like you are lacking in motivation, and would prefer to do nothing? Note that the question isn't referring to your motivation to exercise specifically, but is asking if your attitude more generally to life right now is "I can't be bothered, I'd rather sit and do nothing".

1	2	3	4	5	6	7
Definitely not	Much less than usual	A little less than usual	About average	A bit more than usual	Definitely more than usual	A lot more than usual

7. **I feel sleepy**

Explanation of question: Do you feel like you could easily fall asleep now, or that you would benefit from some sleep if you could?

1	2	3	4	5	6	7
Definitely not	Much less than usual	A little less than usual	About average	A bit more than usual	Definitely more than usual	A lot more than usual

8. **I feel calm**

Explanation of question: Do you feel relaxed and content?

1	2	3	4	5	6	7
Definitely not	Much less than usual	A little less than usual	About average	A bit more than usual	Definitely more than usual	A lot more than usual

9. **I feel distracted**

Explanation of question: Is your head full of many thoughts, and you are having trouble concentrating on one thing?

1	2	3	4	5	6	7
Definitely not	Much less than usual	A little less than usual	About average	A bit more than usual	Definitely more than usual	A lot more than usual

10. **I feel glad**

Explanation of question: Do you feel buoyant at the moment? Are you in a celebratory mood, pleased and happy?

1	2	3	4	5	6	7
Definitely not	Much less than usual	A little less than usual	About average	A bit more than usual	Definitely more than usual	A lot more than usual

Burgess Mood Scale Scoring

Factor analysis by varimax rotation, with polychoric correlations, is applied to the output, which produces two factors:

Drive [Energetic, Alert, Happy/Glad, Focused, Distracted(-1), Sad(-1), Lethargic(-1), Sleepy(-1)], Distracted(-1)

Serenity [Calm, Nervous(-1)]

Reference for previous use of this questionnaire in the context of menstrual cycle effects:

Ronca, F., Blodgett, J.M., Bruinvels, G., Lowery, M., Raviraj, M., Sandhar, G., Symeonides, N., Jones, C., Loosemore, M. and Burgess, P.W., 2025. Attentional, anticipatory and spatial cognition fluctuate throughout the menstrual cycle: Potential implications for female sport. Neuropsychologia, 206, p.108909

Menstrual cycle symptom questionnaire

The menstrual cycle symptom list below was taken from Davison et al. (2022) and Bruinvels et al. (2021), and it was implemented in Ronca et al. (2025) in the current format.

For analysis, symptoms were added up into two categories: Cognitive symptoms and physical symptoms, totalling 14 possible physical symptoms and 8 possible cognitive symptoms.

Are you experiencing any of the following symptoms today?
[Please select all that apply]

**Listed symptom**	**Classification for analysis**
Stomach/menstrual cramps	Physical symptom
Headaches/migraine	Cognitive symptom
Lower back pain	Physical symptom
Joint pain and/or muscle cramps	Physical symptom
Nausea/sickness/vomiting	Physical symptom
Diarrhoea	Physical symptom
Constipation	Physical symptom
Increased tiredness/fatigue	Physical symptom
Dizziness/light-headedness/reduced coordination	Cognitive symptom
Disrupted sleep	Cognitive symptom
Brain fog	Cognitive symptom
Water retention	Physical symptom
Weight gain	Physical symptom
Changes in mood (e.g. stress, anxiety or depression)	Cognitive symptom
Increased irritability	Cognitive symptom
Cravings/increased appetite	Cognitive symptom
Poor concentration/problems remembering	Cognitive symptom
Acne/skin breakouts	Physical symptom
Changes to/difficulties breathing	Physical symptom
Temperature fluctuations	Physical symptom
Bloating/increased gas	Physical symptom
Breast pain/tenderness	Physical symptom

References

Ronca, F., Blodgett, J.M., Bruinvels, G., Lowery, M., Raviraj, M., Sandhar, G., Symeonides, N., Jones, C., Loosemore, M. and Burgess, P.W., 2025. Attentional, anticipatory and spatial cognition fluctuate throughout the menstrual cycle: Potential implications for female sport. Neuropsychologia, 206, p.108909.

Bruinvels, G., Goldsmith, E., Blagrove, R., Simpkin, A., Lewis, N., Morton, K., Suppiah, A., Rogers, J.P., Ackerman, K.E., Newell, J., Pedlar, C., 2021. Prevalence and frequency of menstrual cycle symptoms are associated with availability to train and compete: a study of 6812 exercising women recruited using the Strava exercise app. Br. J. Sports Med. 55 (8), 438–443.

Supplemental Table 1. Reaction times ± standard deviations for the simple reaction time task, sustained attention task and inhibition task for each self-reported athletic level and each phase.

Statistical outputs from ANOVAs are included in Supplemental Table 2 on the next page.

	Menstruation	Follicular	Ovulation	Luteal
	Simple Reaction Time (ms)			
Inactive	327 ± 54	366 ± 94	381 ± 88	366 ± 85
Active	305 ± 52	294 ± 35	291 ± 43	306 ± 55
Competing	332 ± 69	347 ± 136	338 ± 71	307 ± 56
Elite	316 ± 76	358 ± 119	297 ± 35	376 ± 159
	Sustained Attention Reaction Time (ms)			
Inactive	552 ± 86	644 ± 178	557 ± 108	574 ± 93
Active	488 ± 112	471 ± 64	455 ± 65	485 ± 126
Competing	541 ± 121	489 ± 70	486 ± 54	484 ± 85
Elite	532 ± 127	517 ± 94	448 ± 39	604 ± 191
	Inhibition Reaction Time (ms)			
Inactive	407 ± 66	432 ± 82	429 ± 73	438 ± 55
Active	375 ± 60	381 ± 73	360 ± 67	384 ± 76
Competing	429 ± 92	410 ± 85	397 ± 55	402 ± 60
Elite	456 ± 149	457 ± 143	394 ± 62	521 ± 218

Supplemental Table 2. Statistical outputs for ANOVA and contrast analyses for reaction times in the simple reaction time, sustained attention, and inhibition tasks.

**Simple Reaction Time - ANOVA**							
	**Sum of Squares**	**Mean^2 Error**	**NumDF**	**DenDF**	**F**	**p**	**η^2_p**
**Phase**	**80110**	**26703**	**3**	**150**	**4.06**	**.008**	**.02**
**Group**	**88126**	**29375**	**3**	**50**	**4.47**	**.007**	**.16**
**Phase*Group**	**169531**	**18837**	**9**	**150**	**2.87**	**.004**	**.09**

**Main effect of Phase contrasts**	**Estimate**	**SE**	**df**	**t.ratio**	**p**
Follicular – Luteal	-6.44	16.10	150	-.40	.98
Follicular – Menstruation	1.76	16.10	150	.11	.99
**Follicular – Ovulation**	**43.87**	**16.10**	**150**	**2.72**	**.04**
Luteal – Menstruation	8.20	16.10	150	.51	.96
**Luteal – Ovulation**	**50.32**	**16.10**	**150**	**3.12**	**.01**
Menstruation - Ovulation	42.11	16.10	150	2.61	.048

**Main effect of Group contrasts**	**Estimate**	**SE**	**df**	**t.ratio**	**p**
**Inactive – Active**	**106.50**	**29.70**	**50**	**3.59**	**.004**
Inactive – Competing	81.40	34.80	50	2.34	.10
Inactive – Elite	55.80	33.20	50	1.68	.34
Active – Competing	-25.10	31.50	50	-.79	.86
Active – Elite	-50.60	29.70	50	-1.71	.33
Competing – Elite	-25.50	34.80	50	-.73	.88

**Contrasts; Inactive Group**	**Estimate**	**SE**	**df**	**t.ratio**	**p**
Follicular – Luteal	70.02	33.10	150	2.12	.15
**Follicular – Menstruation**	**91.62**	**33.10**	**150**	**2.77**	**.03**
**Follicular – Ovulation**	**86.98**	**33.10**	**150**	**2.63**	**.045**
Luteal – Menstruation	21.60	33.10	150	.65	.91
Luteal – Ovulation	16.96	33.10	150	.51	.96
Menstruation - Ovulation	-4.65	33.10	150	-.14	.99

**Contrasts; Active Group**	**Estimate**	**SE**	**df**	**t.ratio**	**p**
Follicular – Luteal	-13.67	25.60	150	-.53	.95
Follicular – Menstruation	-17.14	25.60	150	-.67	.91
Follicular – Ovulation	16.31	25.60	150	.64	.92
Luteal – Menstruation	-3.47	25.60	150	-.14	.99
Luteal – Ovulation	29.98	25.60	150	1.17	.65
Menstruation - Ovulation	33.45	25.60	150	1.31	.56

**Contrasts; Competing Group**	**Estimate**	**SE**	**df**	**t.ratio**	**p**
Follicular – Luteal	4.84	36.30	150	.13	.99
Follicular – Menstruation	-52.43	36.30	150	-1.45	.37
Follicular – Ovulation	3.12	36.30	150	.09	.99
Luteal – Menstruation	-57.27	36.30	150	-1.58	.39
Luteal – Ovulation	-1.73	36.30	150	-.05	1.00
Menstruation - Ovulation	55.55	36.30	150	1.53	.42

Contrasts; Elite Group	Estimate	SE	df	t.ratio	p
Follicular – Luteal	-86.96	33.10	150	-2.63	.046
Follicular – Menstruation	-15.02	33.10	150	-.45	.97
Follicular – Ovulation	69.09	33.10	150	2.09	.16
Luteal – Menstruation	71.94	33.10	150	2.17	.14
**Luteal – Ovulation**	**156.05**	**33.10**	**150**	**4.72**	**<.001**
Menstruation - Ovulation	84.11	33.10	150	2.54	.06

Contrasts; Follicular Phase	Estimate	SE	df	t.ratio	p
**Inactive – Active**	**172.27**	**39.20**	**129**	**4.39**	**.0001**
**Inactive – Competing**	**154.63**	**46.00**	**129**	**3.36**	**.006**
**Inactive – Elite**	**126.23**	**43.80**	**129**	**2.88**	**.02**
Active – Competing	-17.63	41.60	129	-.42	.97
Active – Competing	-46.04	39.20	129	-1.17	.64
Competing – Elite	-28.41	46.00	129	-.62	.93

Contrasts; Luteal Phase	Estimate	SE	df	t.ratio	p
Inactive – Active	88.56	39.20	129	2.23	.11
Inactive – Competing	89.46	46.00	129	1.95	.21
Inactive – Elite	-30.76	43.80	129	-.70	.90
Active – Competing	.88	41.60	129	.02	1.00
**Active – Elite**	**-119.34**	**39.20**	**129**	**-3.04**	**.02**
**Competing – Elite**	**-120.21**	**46.00**	**129**	**-2.61**	**.049**

Contrasts; Menstruation Phase	Estimate	SE	df	t.ratio	p
Inactive – Active	63.50	39.20	129	1.62	.37
Inactive – Competing	10.58	46.00	129	.23	.99
Inactive – Elite	19.58	43.80	129	.45	.97
Active – Competing	-52.92	41.60	129	-1.27	.58
Active – Elite	-43.92	39.20	129	-1.12	.68
Competing – Elite	9.00	46.00	129	.20	.99

Contrasts; Ovulation Phase	Estimate	SE	df	t.ratio	p
**Inactive – Active**	**101.60**	**39.20**	**129**	**2.59**	**.05**
Inactive – Competing	70.77	46.00	129	1.54	.42
Inactive – Elite	108.34	43.80	129	2.47	.07
Active – Competing	-30.82	41.60	129	-.74	.88
Active – Elite	6.74	39.20	129	.17	.99
Competing – Elite	37.56	46.00	129	.82	.85

Sustained Attention - ANOVA

	Sum of Squares	Mean^2 Error	NumDF	DenDF	F	p	η^2_p
**Phase**	**80110**	**26703**	**3**	**150**	**4.06**	**.008**	**.08**
**Group**	**88126**	**29375**	**3**	**50**	**4.47**	**.007**	**.21**
**Phase*Group**	**169531**	**18837**	**9**	**150**	**2.87**	**.004**	**.15**

Main effect of Phase Contrasts	Estimate	SE	df	t.ratio	p	d_z
Follicular – Luteal	-6.44	16.10	150	-.40	.98	
Follicular – Menstruation	1.76	16.10	150	.11	.99	
**Follicular – Ovulation**	**43.87**	**16.10**	**150**	**2.72**	**.04**	
Luteal – Menstruation	8.20	16.10	150	.51	.96	
**Luteal – Ovulation**	**50.32**	**16.10**	**150**	**3.12**	**.01**	
Menstruation - Ovulation	42.11	16.10	150	2.61	.048	

Main effect of Group Contrasts	Estimate	SE	df	t.ratio	p
Inactive – Active	106.50	29.70	50	3.59	.004
Inactive – Competing	81.40	34.80	50	2.34	.10
Inactive – Elite	55.80	33.20	50	1.68	.34
Active – Competing	-25.10	31.50	50	-.80	.86
Active – Elite	-50.60	29.70	50	-1.71	.33
Competing – Elite	-25.50	34.80	50	-.73	.88

Contrasts; Inactive Group	Estimate	SE	df	t.ratio	p
Follicular – Luteal	70.02	33.10	150	2.12	.15
**Follicular – Menstruation**	**91.62**	**33.10**	**150**	**2.77**	**.03**
**Follicular – Ovulation**	**86.98**	**33.10**	**150**	**2.63**	**.046**
Luteal – Menstruation	21.60	33.10	150	.65	.91
Luteal – Ovulation	16.96	33.10	150	.51	.96
Menstruation - Ovulation	-4.65	33.10	150	-.14	.99

Contrasts; Active Group	Estimate	SE	df	t.ratio	p
Follicular – Luteal	-13.67	25.60	150	-.53	.95
Follicular – Menstruation	-17.14	25.60	150	-.67	.91
Follicular – Ovulation	16.31	25.60	150	.64	.92
Luteal – Menstruation	-3.47	25.60	150	-.14	.99
Luteal – Ovulation	29.98	25.60	150	1.17	.65
Menstruation - Ovulation	33.45	25.60	150	1.31	.56

Contrasts; Competing Group	Estimate	SE	df	t.ratio	p
Follicular – Luteal	4.84	36.30	150	.13	.99
Follicular – Menstruation	-52.43	36.30	150	-1.45	.47
Follicular – Ovulation	3.12	36.30	150	.09	.99
Luteal – Menstruation	-57.27	36.30	150	-1.58	.39
Luteal – Ovulation	-1.73	36.30	150	-.05	1.00
Menstruation - Ovulation	55.55	36.30	150	1/53	.42

Contrasts; Elite Group	Estimate	SE	df	t.ratio	p
**Follicular – Luteal**	**-86.96**	**33.10**	**150**	**-2.63**	**.046**
Follicular – Menstruation	-15.02	33.10	150	-.46	.97
Follicular – Ovulation	69.09	33.10	150	2.09	.16
Luteal – Menstruation	71.94	33.10	150	2.17	.14
**Luteal – Ovulation**	**156.05**	**33.10**	**150**	**4.72**	**<.001**
Menstruation - Ovulation	84/11	33.10	150	2.54	.06

Contrasts; Follicular Phase	Estimate	SE	df	t.ratio	p
**Inactive – Active**	**172.27**	**39.20**	**129**	**4.39**	**.0001**
**Inactive – Competing**	**154.63**	**46.00**	**129**	**3.36**	**.006**
**Inactive – Elite**	**126.23**	**43.80**	**129**	**2.88**	**.02**
Active – Competing	-17.63	41.60	129	-.42	.97
Active – Elite	-46.04	39.20	129	-1.17	.64
Competing – Elite	-28.41	46.00	129	-.62	.93

Contrasts; Luteal Phase	Estimate	SE	df	t.ratio	p
Inactive – Active	88.58	39.20	129	2.26	.11
Inactive – Competing	89.46	46.00	129	1.95	.21
Inactive – Elite	-30.76	43.80	129	-.70	.90
Active – Competing	.88	41.60	129	.02	1.00
**Active – Elite**	**-119.36**	**39.20**	**129**	**-3.04**	**.01**
Competing – Elite	-120.21	46.00	129	-2.61	.048

Contrasts; Menstruation Phase	Estimate	SE	df	t.ratio	p
Inactive – Active	63.50	39.20	129	1.62	.37
Inactive – Competing	10.58	46.00	129	.23	.99
Inactive – Elite	19.58	43.80	129	.45	.97
Active – Competing	-52.92	41.60	129	-1.27	.58
Active – Elite	-43.92	39.20	129	-1.12	.68
Competing – Elite	9.00	46.00	129	.20	.99

Contrasts; Ovulation Phase	Estimate	SE	df	t.ratio	p
**Inactive – Active**	**101.60**	**39.20**	**129**	**2.59**	**.05**
Inactive – Competing	70.77	46.00	129	1.54	.42
Inactive – Elite	108.34	43.80	129	2.47	.07
Active – Competing	-30.82	41.60	129	-.74	.88
Active – Elite	6.74	39.20	129	.17	.99
Competing – Elite	37.56	46.00	129	.82	.85

Inhibition - ANOVA

	Sum of Squares	Mean^2 Error	NumDF	DenDF	F	p	η^2_p
**Phase**	**43417**	**14472.30**	**3**	**150**	**4.31**	**.006**	**.08**
**Group**	**27666**	**9222.10**	**3**	**50**	**2.75**	**.05**	**.14**
**Phase*Group**	**71043**	**7893.70**	**9**	**150**	**2.35**	**.02**	**.12**

**Main effect of Phase Contrasts**	**Estimate**	**SE**	**df**	**t.ratio**	**p**
Follicular – Luteal	-16.30	11.50	150	-1.41	.49
Follicular – Menstruation	3.12	11.50	150	.27	.99
Follicular – Ovulation	24.86	11.50	150	2.16	.14
Luteal – Menstruation	19.42	11.50	150	1.69	.33
**Luteal – Ovulation**	**41.16**	**11.50**	**150**	**3.57**	**.003**
Menstruation - Ovulation	21.74	11.50	150	1.89	.23

**Main effect of Group Contrasts**	**Estimate**	**SE**	**df**	**t.ratio**	**p**
Inactive – Active	51.20	29.50	50	1.73	.32
Inactive – Competing	17.00	34.60	50	.49	.96
Inactive – Elite	-30.60	33.00	50	.93	.79
Active – Competing	-34.20	31.30	50	-1.09	.70
**Active – Competing**	**-81.90**	**29.50**	**50**	**-2.77**	**.04**
Competing – Elite	-47.60	34.60	50	-1.38	.52

**Contrasts; Inactive Group**	**Estimate**	**SE**	**df**	**t.ratio**	**p**
Follicular – Luteal	-5.88	23.60	150	-.25	.99
Follicular – Menstruation	24.16	23.60	150	1.02	.74
Follicular – Ovulation	2.52	23.60	150	.11	.99
Luteal – Menstruation	30.04	23.60	150	1.27	.58
Luteal – Ovulation	8.40	23.60	150	.36	.98
Menstruation - Ovulation	-21.64	23.60	150	-.92	

**Contrasts; Active Group**	**Estimate**	**SE**	**df**	**t.ratio**	**p**
Follicular – Luteal	-3.21	18.30	150	-.18	.99
Follicular – Menstruation	6.16	18.30	150	.34	.99
Follicular – Ovulation	21.08	18.30	150	1.15	.66
Luteal – Menstruation	9.29	18.30	150	.51	.96
Luteal – Ovulation	24.29	18.30	150	1.33	.55
Menstruation - Ovulation	14.92	18.30	150	.82	.85

**Contrasts; Competing Group**	**Estimate**	**SE**	**df**	**t.ratio**	**p**
Follicular – Luteal	8.25	25.90	150	.32	.9
Follicular – Menstruation	-18.32	25.90	150	-.71	.89
Follicular – Ovulation	13.71	25.90	150	.53	.95
Luteal – Menstruation	-26.57	25.90	150	-1.03	.73
Luteal – Ovulation	5.46	25.90	150	.21	.99
Menstruation - Ovulation	32.03	25.90	150	1.24	.60

**Contrasts; Elite Group**	**Estimate**	**SE**	**df**	**t.ratio**	**p**
**Follicular – Luteal**	**-64.35**	**23.60**	**150**	**-2.72**	**.04**
Follicular – Menstruation	.48	23.60	150	.02	1.00
**Follicular – Ovulation**	**62.14**	**23.60**	**150**	**2.63**	**.046**
**Luteal – Menstruation**	**64.83**	**23.60**	**150**	**2.74**	**.03**
**Luteal – Ovulation**	**126.49**	**23.60**	**150**	**5.35**	**<.001**
**Menstruation - Ovulation**	**61.67**	**23.60**	**150**	**2.61**	**.048**

**Contrasts; Follicular Phase**	**Estimate**	**SE**	**df**	**t.ratio**	**p**
Inactive – Active	50.43	34.80	91.30	1.45	.47
Inactive – Competing	21.30	40.80	91.30	.52	.95
Inactive – Elite	-25.00	38.90	91.30	-.64	.92
Active – Competing	-29.13	36.90	91.30	-.79	.86
Active – Elite	-75.43	34.80	91.30	-2.17	.14
Competing – Elite	-46.30	40.80	91.30	-1.14	.67

**Contrasts; Luteal Phase**	**Estimate**	**SE**	**Df**	**t.ratio**	**p**
Inactive – Active	53.09	34.80	91.30	1.53	.43
Inactive – Competing	35.43	40.80	91.30	.87	.82
Inactive – Elite	-83.47	38.90	91.30	-2.15	.15
Active – Competing	-17.67	36.90	91.30	-.48	.96
Active – Elite	-136.56	34.80	91.30	-3.93	.0009
**Competing – Elite**	**-118.90**	**40.80**	**91.30**	**-2.92**	**.02**

**Contrasts; Menstruation Phase**	**Estimate**	**SE**	**df**	**t.ratio**	**p**
Inactive – Active	32.42	34.80	91.30	.93	.79
Inactive – Competing	-21.18	40.80	91.30	-.52	.95
Inactive – Elite	-48.68	38.90	91.30	-1.25	.60
Active – Competing	-53.60	36.90	91.30	-1.45	.47
Active – Elite	-81.11	34.80	91.30	-2.33	.10
Competing – Elite	-27.50	40.80	91.30	-.68	.91

**Contrasts; Ovulation Phase**	**Estimate**	**SE**	**df**	**t.ratio**	**p**
Inactive – Active	68.98	34.80	91.30	.98	.20
Inactive – Competing	32.49	40.80	91.30	.80	.86
Inactive – Elite	34.62	38.90	91.30	.89	.81
Active – Competing	-36.49	36.90	91.30	-.99	.76
Active – Elite	-34.36	34.80	91.30	-.99	.76
Competing – Elite	2.13	40.80	91.30	.05	.99

Supplemental Table 3. Number of errors ± standard deviations for the sustained attention task and inhibition tasks, as well as spatial anticipation timing error (ms) ± standard deviation for each self-reported athletic level and each phase.

Statistical outputs from ANOVAs are included in Supplemental Table 4 on the next page.

	Menstruation	Follicular	Ovulation	Luteal
	Sustained Attention Errors (n)			
Inactive	.50 ± 2.27	2.00 ± 2.19	.50 ± 2.52	3.58 ± 2.19
Active	.20 ± 1.14	.25 ± 1.59	.20 ± 1.83	.25 ± 1.69
Competing	.60 ± 1.60	.20 ± 1.07	.10 ± .82	.01 ± 1.10
Elite	.33 ± 1.78	2.17 ± 1.95	.17 ± .97	.05 ± 1.11
	Inhibition Errors (n)			
Inactive	2.33 ± 2.27	3.67 ± 2.19	3.00 ± 2.52	2.33 ± 2.19
Active	1.35 ± 1.14	1.70 ± 1.59	1.75 ± 1.83	1.70 ± 1.69
Competing	1.10 ± 1.60	1.40 ± 1.07	.70 ± .82	1.10 ± 1.10
Elite	1.58 ± 1.78	1.83 ± 1.95	.75 ± .97	1.17 ± 1.11
	Timing Error (ms)			
Inactive	92 ± 58	108 ± 92	70 ± 23	77 ± 40
Active	62 ± 15	61 ± 14	69 ± 19	64 ± 29
Competing	55 ± 7	62 ± 20	59 ± 10	65 ± 31
Elite	68 ± 30	63 ± 27	62 ± 19	68 ± 27

Supplemental Table 4. Statistical outputs for ANOVA and contrast analyses for sustained attention, and inhibition task errors, as well as timing errors.

Sustained Attention Errors

	Sum of Squares	Mean^2 Error	NumDF	DenDF	F	p	η^2_p
Phase	32.66	10.89	3	150	1.85	.14	.04
**Group**	**60.76**	**20.25**	**3**	**50**	**3.45**	**.02**	**.17**
Phase*Group	82.40	9.16	9	150	1.56	.13	.09

Main effect of Group contrasts	Estimate	SE	df	t.ratio	p	d_z
**Inactive – Active**	**1.42**	**.48**	**50**	**2.99**	**.02**	
Inactive – Competing	1.42	.56	50	2.55	.06	
Inactive – Elite	.85	.53	50	1.61	.38	
Active – Competing	0.00	.50	50	0.00	1.00	
Active – Elite	-.57	.48	50	-1.19	.64	
Competing – Elite	-.57	.56	50	-1.02	.74	

Inhibition Errors

	Sum of Squares	Mean^2 Error	NumDF	DenDF	F	p	η^2_p
**Phase**	**12.70**	**4.23**	**3**	**150**	**3.52**	**.02**	**.07**
**Group**	**12.85**	**2.28**	**3**	**50**	**3.56**	**.02**	**.18**
Phase*Group	16.68	1.85	9	150	1.54	.14	.08

Main effect of Phase contrasts	Estimate	SE	df	t.ratio	p	d_z
**Follicular – Luteal**	**.58**	**.218**	**150**	**2.64**	**.045**	
Follicular – Menstruation	.56	218	150	2.56	.06	
**Follicular – Ovulation**	**.60**	**218**	**150**	**2.75**	**.03**	
Luteal – Menstruation	-.02	218	150	-.08	.99	
Luteal – Ovulation	.03	218	150	.12	.99	
Menstruation - Ovulation	.04	218	150	.19	.99	

Main effect of Group contrasts	Estimate	SE	df	t.ratio	p	d_z
Inactive – Active	1.21	.51	50	2.36	.10	
**Inactive – Competing**	**1.76**	**.60**	**50**	**2.93**	**.03**	
Inactive – Elite	1.50	.57	50	2.62	.06	
Active – Competing	.55	.51	50	1.01	.74	
Active – Elite	.29	.51	50	.57	.94	
Competing – Elite	-.26	.60	50	-.43	.97	

**Timing Errors**							
	**Sum of Squares**	**Mean² Error**	**NumDF**	**DenDF**	**F**	**p**	η^2_p
Phase	1757.40	585.79	3	150	.68	.56	.01
**Group**	**9154.70**	**3051.56**	**3**	**50**	**3.56**	**.02**	**.18**
Phase*Group	10884.20	1209.36	9	150	1.41	.19	.08

**Main effect of Group contrasts**	**Estimate**	**SE**	**df**	**t.ratio**	**p**	d_z
**Inactive – Active**	**22.87**	**8.06**	**50**	**2.84**	**.03**	
**Inactive – Competing**	**26.40**	**9.45**	**50**	**2.79**	**.04**	
Inactive – Elite	21.37	9.01	50	2.37	.10	
Active – Competing	3.53	8.55	50	.41	.98	
Active – Elite	-1.50	8.06	50	-.19	.99	
Competing – Elite	-5.03	9.45	50	-.53	.95	

Supplemental Table 5. Number of participants (n = 41) reporting experiencing each symptom at each phase.

Symptom	**Early follicular**	**Late follicular**	**Ovulation**	**Mid-luteal**
Brain fog	10	5	5	8
Cravings	14	2	2	7
Dizziness or poor coordination	11	1	3	3
Irritability	13	1	2	6
Migraine or headache	10	6	5	5
Disrupted sleep	14	6	8	13
Poor concentration or memory	10	5	5	9
Stress, anxiety or depression	14	5	6	8
Cognitive symptoms (any of the above)	**34**	**21**	**18**	**25**
Acne	9	9	4	11
Bloating	15	2	5	0
Breast pain	5	0	4	4
Changes to breathing	1	0	0	2
Constipation	3	0	0	0
Cramps	33	0	2	1
Diarrhoea	6	1	3	1
Fatigue	21	10	9	15
Joint or muscle pain	5	2	1	1
Lower back pain	20	3	2	2
Nausea	3	0	4	1
Temperature fluctuations	7	2	2	2
Water retention	9	2	5	2
Weight gain	5	2	3	3
Physical symptoms (any of the above)	**39**	**21**	**21**	**25**
No Symptoms	**0**	**17**	**16**	**10**
